# Blood meal profile and positivity rate with malaria parasites among different malaria vectors in Sudan

**DOI:** 10.1186/s12936-022-04157-y

**Published:** 2022-04-15

**Authors:** Omnia Altahir, Hanadi AbdElbagi, Mustafa Abubakr, Emmanuel Edwar Siddig, Ayman Ahmed, Nouh Saad Mohamed

**Affiliations:** 1 Molecular Biology Unit, Sirius Training and Research Centre, Khartoum, Sudan; 2 Department of Epidemiology, Tropical Medicine Research Institute, National Centre for Research, Khartoum, Sudan; 3 Department of the Integrated Vector Management (IVM), Federal Ministry of Health, Khartoum, Sudan; 4 Mycetoma Research Center, University of Khartoum, Khartoum, Sudan; 5 Department of Cytology and Histopathology, Faculty of Medical Laboratory Sciences, University of Khartoum, Khartoum, Sudan

**Keywords:** Blood meal source, Malaria parasites, Mosquito vectors, Sudan

## Abstract

**Background:**

Malaria is a life-threatening public health problem globally with particularly heavy burden in the sub-Saharan Africa including Sudan. The understanding of feeding preference of malaria vectors on different hosts is a major challenge for hindering the transmission cycle of malaria. In this study, blood meals taken by blood-fed *Anopheles* mosquitoes collected from the field in malaria endemic areas of Sudan were analysed for source of blood meal and malaria parasite presence.

**Methods:**

*Anopheles* mosquitoes were collected from different regions in Sudan: Khartoum state, Sennar state, Northern state, and El Gedarif state between September 2020 and February 2021. *Anopheles* mosquitoes were collected using the standard pyrethrum spray catch and back-pack aspirator. Mosquito samples were sorted and morphologically identified to species level using international identification keys. Morphologically identified mosquito species were also confirmed using PCR. Genomic DNA was extracted from mosquitoes for molecular identification of blood meal source and parasite detection. The presence of *Plasmodium* species DNA in each mosquito sample was investigated using semi-nested PCR. Frequency of each blood meal source, *Anopheles* mosquito vector, and malaria parasite detected was calculated. Positivity rate of each fed female *Anopheles* mosquito was calculated for each species.

**Results:**

A total of 2132 *Anopheles* mosquitoes were collected. 571 (26.8%) were males and 1561 (73.2%) were females classified based on their abdominal status into 1048 (67.1%) gravid, 274 (17.6%) fed, and 239 (15.3%) unfed females. Among the blood fed *Anopheles* mosquitoes, 263 (96.0%) were morphologically identified and confirmed using PCR to *Anopheles arabiensis*, 9 (3.3%) to *Anopheles stephensi*, and 2 (0.7%) to *Anopheles rufipes*. Of 274 blood-fed *An. arabiensis*, 68 (25.9%) fed on mixed blood meals from human and cattle, 8 (3.0%) fed on cattle and goat, and 13 (4.8%) fed on human, cattle and goat. For single blood meal sources, 70 (26.6%) fed on human, 95 (36.1%) fed on cattle, 8 (3.0%) fed on goat, and 1 (0.4%) fed on dog. While *An. rufipes* and *An. stephensi* fed on dog (2; 0.75%) and cattle (9; 3.3%), respectively. *Plasmodium* parasite detection in the blood meals showed that 25/274 (9.1%) *An. arabiensis* meals were positive for *Plasmodium vivax* and 19/274 (6.9%) *An. arabiensis* meals were positive for *Plasmodium falciparum*. The rate of positivity of *An. arabiensis* with any *Plasmodium* species was 16.7%. However, the positivity rate with *P. falciparum* only was 7.2%, while *P. vivax* was 9.5%. Both *An. rufipes* and *An. stephensi* were having positivity rates of 0.0% each.

**Conclusions:**

This study which was mainly on blood-fed *Anopheles* mosquitoes showed a diversity in the type of diet from human, cattle, and goat. *Anopheles* mosquitoes especially *An. arabiensis* in Sudan, are opportunistic blood feeders and can feed broadly on both human and cattle. The application of blood meal identification is not only important in malaria vector epidemiological surveillance but also is very useful in areas where arthropods exhibit zoophilic feeding behaviour for mammals.

## Background

Vector-borne diseases (VBDs) are transmitted by arthropod vectors when they feed on vertebrate host blood [1]. The role of a disease vector in the transmission of VBDs largely depends on its host preference [2]. Feeding on a different host and the host preference of the diseases’ vectors constitute a significant challenge for transmitting zoonotic diseases that infect both human and animal populations. These include leishmaniasis, onchocerciasis, and arboviral diseases [3–6]. Thus, a broader range of hosts’ availability as sources of blood meals contributes substantially to the diseases’ transmission [2]. Malaria is a life-threatening public health problem globally with particularly heavy burden in the sub-Saharan African region including Sudan with frequent outbreaks [7].

Mosquitoes feed on a wide range of vertebrate hosts, including amphibians, reptiles, birds, and mammals [8–11]. The behaviour of targeting a single species is not the choice of blood-feeding mosquitoes. Despite the trait of host preference being innate and controlled by genes, it is affected by confounding factors, such as host availability and accessibility [12]. The vector’s choice of host is important as it affects host/pathogen relationship, which may differ accordingly, ranging from a pathogen with a wide range of susceptible hosts to a less vector-host specificity [13]. Host feeding preference affects the vectorial capacity of mosquitoes and vectors control programme.

Globally, there were an estimated 241 million malaria cases and 627 thousand malaria deaths in 2021, with more than 95% of the cases being reported in Africa [14]. The main malaria vectors in Africa belong to three major groups of vectors, the *Anopheles gambiae* complex, the *Anopheles funestus* group, and the *Anopheles nili* complex [15, 16]. However, recently the invasive Asian malaria vector, *Anopheles stephensi* has emerged in the Horn of Africa region and is rapidly spreading in the area [17–20].

With 2 million cases and 5,000 deaths, malaria is a disease of serious public health importance in Sudan [21]. The major mosquito vector species is *Anopheles arabiensis* [22], but other species, such as *An. funestus*, *Anopheles pharoensis* and *An. nili*, have been identified [23, 24]. *Anopheles stephensi* is increasingly spreading throughout the country [18, 19].

Different assays have been used to identify the blood meal sources in mosquitoes, such as multiplex PCR [25, 26], microsatellites [27], enzyme-linked immunosorbent assay (ELISA) or precipitin test [28, 29]. In this study, blood meals taken by blood-fed mosquitoes collected in the field from different areas of Sudan were analysed to identify the source of blood meal and the presence of malaria parasite using multiplex PCR.

## Methods

### Samples collection and study areas

**Fig. 1:**
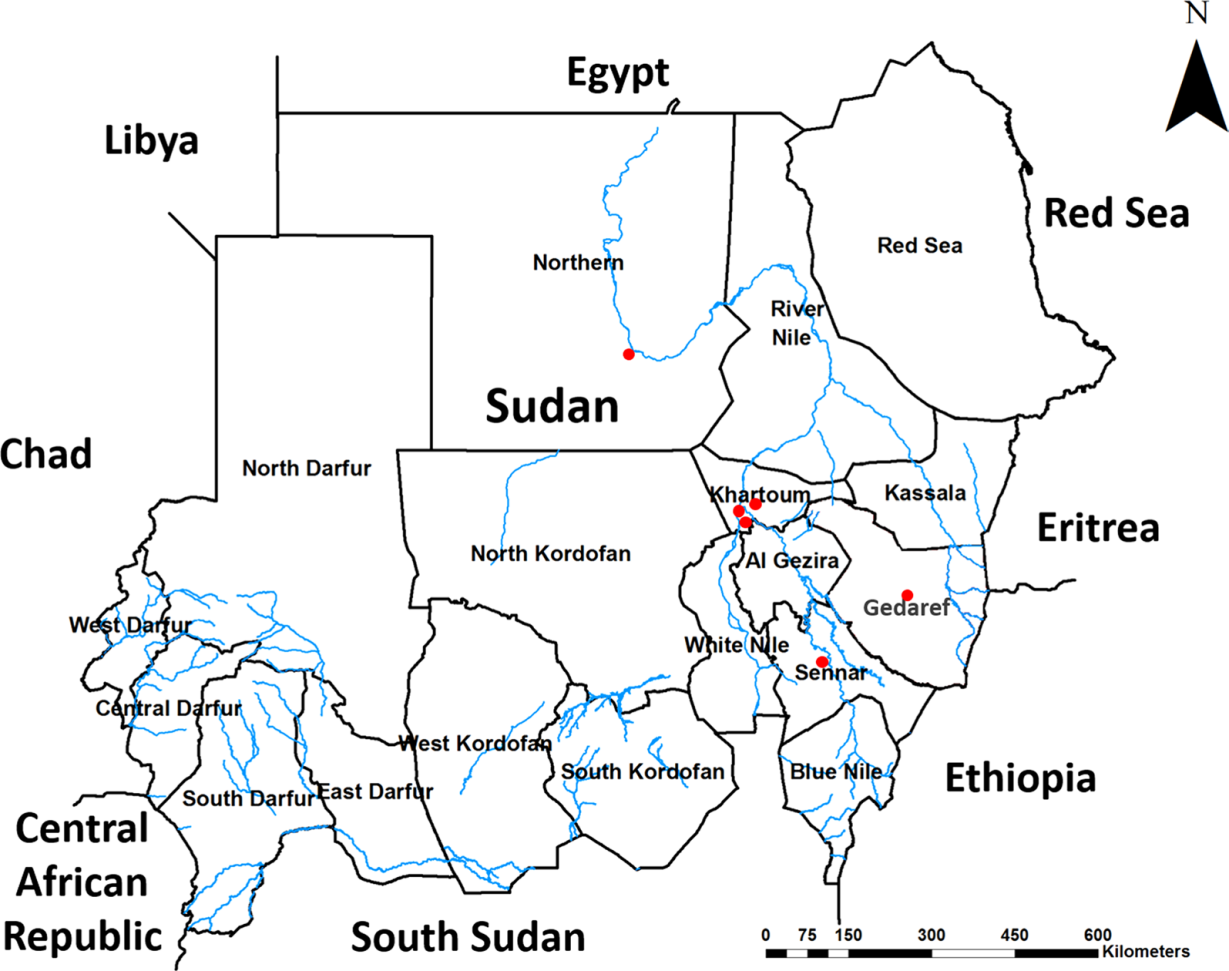
Sudan map. Showing the study areas where mosquito samples were collected. The red dots represent each site location

Wild blood-fed samples of *Anopheles* were collected from different regions in Sudan, namely; Khartoum state (15°55′N 32°53′E), Northern state (19° 37′ N 29° 38′ E), Al Gedarif state (14° 02′ N 35° 23′ E), and Sennar state (12° 58′ N 34° 3′ E) (Fig. 1). These regions were considered as mesoendemic areas with different malaria seasonality [30]. In the studied areas, *Plasmodium falciparum* is the most common malaria parasite, responsible for 90% of malaria infections, while 10% are caused by *Plasmodium vivax* [31]. *Anopheles* collection was carried out simultaneously inside the rooms of 20 houses in each study site for five consecutive days. *Anopheles* samples were collected using the standard pyrethrum spray catch (PSC) and back-pack aspirator. The collected samples were morphologically identified to species level using international identification keys and sorted out microscopically according to their sex and status of blood feeding [32, 33]. *Anopheles* males, and unfed and gravid females were excluded from the blood meal analysis due to the lack of feeding on blood for the formers and the low yield of blood source DNA for the later [25]. *Anopheles* samples were then preserved with silica gel until DNA extraction.

### DNA extraction

Genomic DNA was extracted from mosquitoes individually using sodium chloride-Tris-EDTA (STE) solution following the method of Kent and Norris [25] with a minor modification. Briefly, each mosquito was placed into 100 µl STE, crushed in 1.5 ml Eppendorf tube using glass pestle until complete homogenization. Homogenized samples were then incubated at 65° C for a single hour. Following the incubation, 30 µl of potassium acetate 8.0 M was added and then placed at − 20 °C for 1 h. After freezing, samples were allowed to thaw at room temperature and then centrifuged for 15 min at 15,000 rpm. The supernatant was then transferred into a new tube and 1 ml of absolute ethanol was added to precipitate the DNA. Samples were then placed at − 20 °C for 30 min to increase DNA precipitation. Then, the samples were subjected to high-speed centrifugation at 15,000 rpm for 10 min. The supernatant was discarded and the pellet was allowed to dry completely. Dried pellets were then dissolved using 30 µl of deionized distilled water. Extracted DNA was checked for purity and concentration using nanodrop (Implen, Germany) and preserved at − 20 °C until molecular identification of blood meal source and parasite detection.

### Molecular confirmation of the species identity

Individual mosquitoes that morphologically identified as *An. gambiae sensu lato* (*s.l*.) were further investigated using PCR. Maxime™ i-Taq PCR premix kit was used to prepare the PCR reaction mixture according to the manufacturer instructions (iNtRON Biotechnology, South Korea). Primers and cycling condition used for the confirmation of the *An. gambiae s.l.* were as previously shown [34]. The PCR reactions were performed using 2027 Thermocycler (Applied Biosystems, Germany). The specific primers of each species produce a band size of 315 in case of *An. arabiensis*, and a product size of 395 bp in case of *An. gambiae*.

### Molecular identification of the blood meal

Sources of blood meals were identified from the extracted DNA using the previously published primers [25, 35, 36]. To identify blood meal source of human, cattle, goat, dog, bird, and reptile, the following primers were used; Human-F: 5′ CCT ACT CCT GCT CGC ATC TG ‘3 and Human-R: 5′ AGA ATG GGG TCT CCT CCT CC ′3, Cattle-F: 5’ CCC ATC CTA TTG GCC GTA GC ′3, Cattle-R: 5’ GAT GTA GCG GGT CGT AGT GG ‘3, Goat-F: 5’ ACG TAG AAT ATG CCG CAG GG ‘3, Goat-R: 5’ CGT AAC GGA ATC GGG GGT AG ‘3, Dog-F: 5’ GCC TTC CTG ACC CTT GTT GA ‘3, Dog-R: 5’ TTA CTG CGT CTG CGA TTG GT ‘3, Bird-F: 5’ CGC CTG TTT ATC AAA AAC AT ′3, Bird-R: 5′ CCG GTC TGA ACT AGA TCA CGT ‘3, Reptile-F: 5’ TNT TMT CAA CNA ACC ACA AAG A ‘3, and Reptile-R: 5’ ACT TCT GGR TGK CCA AAR AAT CA ‘3 [36]. The PCR reaction mixture to identify the blood meal source was in total of 25 µl containing 10 mM Tris-HCl (pH 8.3), 2 mM MgCl_2_, 200 µM of each dNTP, 0.1 U Taq polymerase (i-Taq Plus™, DNA Polymerase, iNtRON Biotechnology, South Korea). Adding to 21 µl of the PCR reaction mixture, 1 µl 10 pmol of each of the forward and reverse primers of each source separately and 2 µl of the extracted DNA and incubated in the PCR amplification condition as followed, an initial denaturation step at 95° C for 5 min followed by 14 cycles of denaturation at 95 °C for 30 s, annealing at 60 °C for 30 s with decrement of 0.5 °C each cycle, and extension at 72 °C for 30 s followed by another 19 cycles of denaturation at 95 °C for 30 s, annealing at 53 °C for 30 s, and extension at 72 °C for 30 s. Finally, a final extension step at 72 °C for 10 min and then the PCR products were cooled down to 4 °C. Positive DNA samples of human, cattle (*Bos taurus*), goat (*Capra hircus*), dog (*Canis familiaris*), bird (*Columba livia domestica*), and reptile (*Hemidactylus species*) were used as positive controls for each specific primer, while double distilled water was used as a negative control in each run. Molecular identification of blood meal was interpreted according to the specific band sizes produced for each blood meal primers. Blood meals of human, cattle, goat, and dog sources were presented with PCR product sizes of 363 bp, 164 bp, 213 bp, and 109 bp, respectively.

### Molecular detection of ***Plasmodium*** species

To investigate the positivity rate of *Anopheles* with malaria parasites, the presence of the DNA of *Plasmodium* species in each mosquito sample was investigated. Using the semi-nested PCR, the primers used for the detection of the 4 human malaria parasites in Sudan including *P. falciparum, P. vivax, Plasmodium ovale*, and *Plasmodium malariae* were adopted from Rubio et al. [37] including; UN-R: 5′ GAC GGT ATC TGA TCG TCT T 3′, UN-F: 5′ AGT GTG TAT CAA TCG AGT TT 3′, for the first PCR reaction and UN-F primer and Fal-R: 5′ AGT TCC CCT AGA ATA GTT ACA 3′, Viv-R: 5′ AGG ACT TCC AAG CCG AAG 3′, Ova-R: 5′ GCA TAA GGA ATG CAA AGA ACA G 3′, and Mal-R: 5′ GCC CTC CAA TTG CCT TCT 3′, for the second PCR reaction to confirm the presence of *Plasmodium* species. PCR cycling condition was adjusted according to Mohamed et al. [38]. Positive DNA of *P. falciparum, P. vivax, P. ovale*, and *P. malariae* were used as positive controls in each PCR run. Double distilled water was used as a negative control.

### Gel electrophoreses and amplicons interpretation

Following PCR amplification, PCR products were visualized using 2.5% agarose gel. Electrophoresis was made in comparison to a 100 bp DNA ladder in 5X Tris Borate EDTA running buffer in 100 V and 25 A for 1 h. Lengths of amplicons were read by placing the agarose gel on UV-transilluminator (Major Sciences, USA).

### Statistical analysis

Data analysis of the blood meal and parasite positivity rate was made using the Statistical Package for Social Sciences (SPSS version 20.0). Frequency of each blood meal source, *Anopheles* vector species, and malaria-positive mosquitoes was calculated. Positivity rate of each fed female *Anopheles* was calculated for each species by dividing the number of blood meals detected positive for parasite presence on the total number of mosquitoes belonging to that species. Chi-Square test was calculated to test the significance association of *Anopheles* blood meal sources with host preference, and the presence of *Plasmodium* species. A P value less than 0.05 was considered statistically significant.

## Results

### Distribution, morphological and molecular identification of ***Anopheles*** mosquitoes

A total of 2132 *Anopheles* were collected. Of these, 571 (26.8%) were males and 1561 (73.2%) were females. Of the 1561 female *Anopheles*, 1048 (67.1%) were gravid, 274 (17.6%) were blood fed and 239 (15.3%) were blood unfed. Based on site of collection, Sennar state constituted the majority of the collected samples; 1126 (52.8%), while El Gedarif contributed the least collected samples; 86 (4.0%) (Table 1). Of the 274 fed *Anopheles* females, 263 (96.0%) were *An. arabiensis*, 9 (3.3%) were *An. stephensi*, and 2 (0.7%) were *Anopheles rufipes*. The molecular confirmation of *An. arabiensis* was confirmed by the presence of a PCR product band size of 315 bp. According to site of collection, *An. arabiensis* was found in all the study sites except in El Gedarif in which only 9 fed females of *An. stephensi* were collected. The 2 (0.7%) collected *An. rufipes* fed females were found in Sennar state (Table 2).

**Table 1:** The distribution of *Anopheles* mosquitoes collected in the study sites

Sample Location	Male	Gravid Female	Unfed Female	Fed Female	Total
El Gedarif state	23 (26.7%)	54 (62.8%)	0 (0.0%)	9 (10.5%)	86 (4.0%)
Khartoum state	242 (40.2%)	237 (39.4%)	59 (9.8%)	64 (10.6%)	602 (28.3%)
Northern state	87 (27.4%)	179 (56.2%)	20 (6.3%)	32 (10.1%)	318 (14.9%)
Sennar state	219 (19.4%)	578 (51.3%)	160 (14.3%)	169 (15.0%)	1126 (52.8%)
Total	571 (26.8%)	1048 (49.1%)	239 (11.2%)	274 (12.9%)	2132 (100%)

**Table 2:** The distribution of the wild caught *Anopheles* mosquitoes from the different study sites

Sample Location	Vector Identification			Total
	*An. arabiensis*	*An. rufipes*	*An. stephensi*	
El Gedarif state	0 (0.0%)	0 (0.0%)	9 (100%)	9 (3.3%)
Khartoum state	64 (100%)	0 (0.0%)	0 (0.0%)	64 (23.4%)
Northern state	32 (100%)	0 (0.0%)	0 (0.0%)	32 (11.8%)
Sennar state	167 (98.8%)	2 (1.2%)	0 (0.0%)	169 (61.5%)
Total	263 (96.0%)	2 (0.7%)	9 (3.3%)	274 (100%)

### Molecular identification of blood meals sources

A total of 193 (70.4%) blood-fed *Anopheles* fed on cattle, 151 (55.1%) fed on human, 29 (10.6%) fed on goat, and 3 (1.1%) fed on dog. There were no mosquitoes with blood meals from birds or reptiles. When illustrating the source of blood meal based on multiple or single source, multiple blood meals of human and cattle, cattle and goat, and human, cattle and goat were detected; 68 (24.8%), 8 (2.9%), and 13 (4.7%), respectively. Also, single blood meal source was detected among 70 (25.5%) fed on human, 104 (38.0%) fed on cattle, 8 (2.9%) fed on goat, and 3 (1.1%) fed on dog. According to species stratification, the two *An. rufipes* fed on dogs and the nine *An. stephensi* fed on cattle. The association of blood meal source with the collected *Anopheles* mosquitoes was found to be statistically significant for meals detected from human, cattle, and goat, P values; 0.001, 0.014, and 0.001, respectively (Table 3).

**Table 3:** The association of *Anopheles* mosquitoes blood meal sources with host preference

Meal source	Vector identification			Total	P value
	*An. arabiensis*	*An. rufipes*	*An. stephensi*		
Human	151 (57.4%)	0 (0.0%)	0 (0.0%)	151 (55.1%)	0.001
Cattle	184 (70.0%)	0 (0.0%)	9 (100%)	193 (70.4%)	0.014
Goat	29 (11.0%)	0 (0.0%)	0 (0.0%)	29 (10.6%)	0.508
Dog	1 (0.4%)	2 (100%)	0 (0.0%)	3 (1.1%)	0.001
Bird	0 (0.0%)	0 (0.0%)	0 (0.0%)	0 (0.0%)	0.986
Reptile	0 (0.0%)	0 (0.0%)	0 (0.0%)	0 (0.0%)	0.986

### ***Plasmodium*** species detection and the rate of mosquitoes’ positivity

The results of molecular detection of *Plasmodium* species showed the presence of *Anopheles* mosquitoes infected with *P. falciparum* and *P. vivax*. However, none of the samples harboured *P. ovale* or *P. malariae*. Also, no *Plasmodium* species co-infection was detected. *Plasmodium* parasite detection in the blood meals showed that 25 (9.1%) were harbouring *P. vivax* and 19 (6.9%) were harbouring *P. falciparum*. The remaining 230 (83.9%) were found negative. All the *P. falciparum* and *P. vivax* detected were only present in blood meals of *An. arabiensis*. *Plasmodium falciparum* positive blood meals reported from cattle and goat mixed meals were 8 (42.1%) and 3 (15.8%), respectively. Also, among the single blood meal of human, *P. vivax* infection was detected; 12 (48.0%). However, the presence of *P. vivax* among multiple blood meals from human, cattle and goat was 13 (52.0%). In contrast, *P. falciparum* was noted among multiple blood meals of cattle and goat; 8 (42.1%) (Table 4).

**Table 4:** Blood meals sources detected among the wild caught *Anopheles* mosquitoes

Vector identification	Meal Source							Total
	Human, cattle, and goat	Human and cattle	Cattle and goat	Human	Cattle	Goat	Dog	
*An. arabiensis*	13 (4.8%)	68 (25.9%)	8 (3.0%)	70 (26.6%)	95 (36.1%)	8 (3.0%)	1 (0.4%)	263 (96.0%)
*An. rufipes*	0 (0.0%)	0 (0.0%)	0 (0.0%)	0 (0.0%)	0 (0.0%)	0 (0.0%)	2 (100%)	2 (0.7%)
*An. stephensi*	0 (0.0%)	0 (0.0%)	0 (0.0%)	0 (0.0%)	9 (100%)	0 (0.0%)	0 (0.0%)	9 (3.3%)
Vector Infectivity rate								
*An. arabiensis*								
*P. falciparum*	0 (0.0%)	0 (0.0%)	8 (42.1%)	0 (0.0%)	8 (42.1%)	3 (15.8%)	0 (0.0%)	19 (7.2%)
*P. vivax*	13 (52.0%)	0 (0.0%)	0 (0.0%)	12 (48.0%)	0 (0.0%)	0 (0.0%)	0 (0.0%)	25 (9.5%)
Negative	0 (0.0%)	68 (31.0%)	0 (0.0%)	58 (26.5%)	87 (39.7%)	5 (2.3%)	1 (0.5%)	219 (83.3%)
Total	13 (4.9%)	68 (25.9%)	8 (3.0%)	70 (26.6%)	95 (36.1%)	8 (3.0%)	1 (0.4%)	263 (96.0%)
*An. rufipes*								
*P. falciparum*	0 (0.0%)	0 (0.0%)	0 (0.0%)	0 (0.0%)	0 (0.0%)	0 (0.0%)	0 (0.0%)	0 (0.0%)
*P. vivax*	0 (0.0%)	0 (0.0%)	0 (0.0%)	0 (0.0%)	0 (0.0%)	0 (0.0%)	0 (0.0%)	0 (0.0%)
Negative	0 (0.0%)	0 (0.0%)	0 (0.0%)	0 (0.0%)	0 (0.0%)	0 (0.0%)	2 (100%)	2 (100%)
Total	0 (0.0%)	0 (0.0%)	0 (0.0%)	0 (0.0%)	0 (0.0%)	0 (0.0%)	2 (100%)	2 (0.7%)
*An. stephensi*								
*P. falciparum*	0 (0.0%)	0 (0.0%)	0 (0.0%)	0 (0.0%)	0 (0.0%)	0 (0.0%)	0 (0.0%)	0 (0.0%)
*P. vivax*	0 (0.0%)	0 (0.0%)	0 (0.0%)	0 (0.0%)	0 (0.0%)	0 (0.0%)	0 (0.0%)	0 (0.0%)
Negative	0 (0.0%)	0 (0.0%)	0 (0.0%)	0 (0.0%)	9 (100%)	0 (0.0%)	0 (0.0%)	9 (100%)
Total	0 (0.0%)	0 (0.0%)	0 (0.0%)	0 (0.0%)	9 (100%)	0 (0.0%)	0 (0.0%)	9 (3.3%)
Total	13 (4.7%)	68 (24.8%)	8 (2.9%)	70 (25.5%)	104 (38.0%)	8 (2.9%)	3 (1.1%)	274 (100%)

The positivity rate of *An. arabiensis* with either *P. falciparum* and *P. vivax* was 16.7% (44/263). However, the positivity rate with *P. falciparum* was 7.2% (19/263), while positivity rate with *P. vivax* was 9.5% (25/263). Both *An. rufipes* and *An. stephensi* were having positivity rates of 0.0% each (Table 4). The presence of *Plasmodium* parasites among the different meal sources was statistically significant among meals of human, cattle, and goat, P values 0.001, 0.052, 0.001, respectively. Although 3 (1.1%) dog meals were detected, none of them showed presence of *Plasmodium* species, P value 0.748 (Table 5).

**Table 5:** Explication of the detected *Plasmodium* species detected in the blood meals of the wild caught *Anopheles* mosquitoes

	Parasite detection			Total	P value
	*P. falciparum*	*P. vivax*	Negative		
Human	0 (0.0%)	25 (16.6%)	126 (83.4%)	151 (55.1%)	0.001
Cattle	16 (8.3%)	13 (6.8%)	164 (84.9%)	193 (70.4%)	0.052
Goat	11 (37.9%)	13 (44.8%)	5 (17.3%)	29 (10.6%)	0.001
Dog	0 (0.0%)	0 (0.0%)	3 (100%)	3 (1.1%)	0.748
Bird	0 (0.0%)	0 (0.0%)	0 (0.0%)	0 (0.0%)	na
Reptile	0 (0.0%)	0 (0.0%)	0 (0.0%)	0 (0.0%)	na

## Discussion

Identifying the source of mosquito blood meals is of paramount importance in malaria epidemiological studies [39]. The correct identification of the preferred host for malaria vectors determines the major hosts in the area that support the sustainability of vector population. It is also useful in the estimation of vectorial capacity and identifying the role of each mosquito species in malaria transmission [40]. Studies on feeding behaviour of *Anopheles* mosquitoes have been implemented in Kenya [41], Ethiopia [42], Mali [25], Sri Lanka [26], and Sudan [43]. In this study, blood meal sources of human, cattle, goat, dog have been identified. The low prevalence of fed females (12.9%) reported in this study compared to gravid females (49.1%), supports the assumption that mosquito collection for blood meal analysis is mainly affected by the resting position especially after taking the blood meal [42]. Recently, a study conducted by Finney et al. indicated that most of the human blood meals were from *Anopheles* mosquitoes trapped outdoors, while many livestock blood meals were from *Anopheles* mosquitoes trapped indoors [44].

The abundance of *An. arabiensis* in Sennar compared to the other sites was similar to a previous study in which the majority of the collected mosquitoes were *An. arabiensis*; 88.5% [45]. This difference in mosquito abundance can be explained by interference of many factors such as seasonality and the availability of suitable resources for survival in the environment [46].

The results of molecular analysis of blood meal source revealed that *Anopheles* feeding from cattle; 70.4%, was relatively higher than feeding from human; 55.1%, and higher compared to other hosts investigated, including goat and dog; 10.6% and 1.1%, respectively. These results disagree with a previous report from Burkina Faso, where human blood meal source was more than 80% [47]. However, this variation can be as a result of host availability in that specific region [44].

The malaria parasite detected in this study shows the importance of mosquito blood meal analysis in terms of determination of parasite reservoir host. Although, this assumption is not fully supported by this study, either through animal screening or sporozoite detection in the salivary glands of the infected vector. However, it can hint to the need for a wide animal screening especially during the non-transmission seasons of malaria [44]. These results should be interpreted with caution. It has been reported previously [25] that identification of the source of blood meals in mosquitoes was only possible up to 48 h post-feeding; excluding gravid females from the analysis could, therefore, have been one limitation of this study that could have affected frequencies of blood meals source detected.

The chances of *Anopheles* to encounter the parasite in the blood meal of infected human can be very high during the peak transmission season [30, 38, 47]. This can affect significantly the role of vector population in transmitting malaria, such as described by Guelbéogo et al. [47], who reported a high frequency of *Anopheles coluzzii* engorged females with human-animal mixed blood meals. The ability of *Anopheles* to feed on multiple hosts has been also reported previously [48–50]. In this study, the results of multiple blood meals detected are similar to reports from Ethiopia, where multiple feeding on human and cattle was present when both hosts share the same house or closely present in the area [51]. Furthermore, the results of *An. rufipes* and *An. stephensi* feeding on dog and cattle, indicate that a wide range of host preference can occur in Sudan. This also emphasizes a need for wider-scale studies in which *An. stephensi* can be studied extensively, since no previous studies of *An. stephensi* feeding behaviour has been reported [17, 52].

The positivity rate of *An. arabiensis* with *P. falciparum* and *P. vivax* reported in this study has similarities to mosquito positivity rates reported from Burkina Faso, where mosquito positivity rates during the period of malaria transmission season were 5.1%, 13.9%, 6.5%, during the start, the peak, and the end of transmission season, respectively [47]. Although, the study period was performed during the transmission season, the calculated positivity rate might be confounded by the time of collection. Higher positivity rates can be seen during the transmission season where chances of *Anopheles* mosquitoes to encounter the parasite is much higher. Although, no *Plasmodium* species co-infection was detected and all the detected *P. falciparum* and *P. vivax* were only present in blood meals of *An. arabiensis*. This is worrying since the presence of one of the *Plasmodium* species in a vector leads to increase vector ability to multiple feeding on hosts [53]. Further, the introduction of *An. stephensi* since 2016 is quite alarming since its known to be a notorious vector in transmitting both *P. falciparum* and *P. vivax*, as well as being capable of transmitting zoonotic malaria parasites, such as *Plasmodium knowlesi* [54]. This urges for improving the vector control strategies in any area with change in the vector composition or change in the vector behaviour [17].

The most prevalent fed *Anopheles* in this study was *An. arabiensis* compared to *An. rufipes* and *An. stephensi*; although, this can be due to relative vector abundance, it might also be explained by the variation in mosquito survival and age and their relation with malaria transmission. In a similar scenario, the abundance *of An. gambiae sensu stricto* (*s.s*.) was linked to its importance for malaria transmission. In contrast, longevity was lower for other species with less contribution to malaria transmission leading to infrequent capture during vector surveillance [47]. Additionally, the contribution of other *Anopheles* species in transmitting malaria depends on the successful of the *Anopheles* species to inoculate the parasite in more than one host through multiple feeding [55, 56]. All this together, shows the importance of using blood meal analysis escorted with assessment of mosquito positivity rate in malaria epidemiological studies can significantly improve the understanding of identifying specific reservoirs, which harbour the parasite until the next malaria transmission season.

## Conclusions

This study which was mainly on blood-fed *Anopheles* mosquitoes showed a diversity in the type of diet from human, cattle and goat. With the exclusion of the three blood meal samples from dogs, most analysed blood meals were identified as cattle, human, and goat. This study might give the assumption that *Anopheles* mosquitoes in Sudan, especially *An. arabiensis*, are intrinsic unprincipled feeders and can feed broadly on both humans and cattle. However, these results need to be considered carefully given the fact that limitation of the study on the active biting ratio analysis was not applicable. These findings might also indicate that humans, cattle, and goat were only the most abundant host species exists, and this by any means does not inevitably mean that humans and cattle are the preferred hosts in Sudan. Also, the application of blood meal identification is not only important in malaria vector epidemiological surveillance but also is very useful in areas where arthropods exhibit zoophilic feeding behaviour for mammals, such as Rift Valley fever virus where mosquito vectors affect cattle and humans.

## Data Availability

All datasets generated for this study are included in the manuscript.

## Abbreviations

DNA: Deoxyribonucleic acid
EDTA: Ethylene diamine tetra acetic acid
PCR: Polymerase chain reaction
STE: Sodium chloride-Tris-EDTA
